# Enhanced Pain Reduction at Different Stages of Knee Osteoarthritis via Repeated Injections of Hyaluronic Acid with Niacinamide: A Comparative Study

**DOI:** 10.3390/jcm13247553

**Published:** 2024-12-12

**Authors:** Sophie Pennekamp, Stephan Hegelmaier, Wolfgang Hitzl, Markus Geßlein, Hermann Josef Bail, Kim Loose, Andreas Kopf, Niklas Engel, Johannes Rüther, Maximilian Willauschus, Michael Millrose

**Affiliations:** 1 Department of Orthopedics and Traumatology, Paracelsus Medical University, Breslauer Strasse 201, 90471 Nuremberg, Germany; stephan.hegelmaier@stud.pmu.ac.at (S.H.); markus.gesslein@klinikum-nuernberg.de (M.G.); hermann-josef.bail@klinikum-nuernberg.de (H.J.B.); kim.loose@klinikum-nuernberg.de (K.L.); andreas.kopf@klinikum-nuernberg.de (A.K.); niklas.engel@klinikum-nuernberg.de (N.E.); johannes.ruether@klinikum-nuernberg.de (J.R.); maximilian.willauschus@klinikum-nuernberg.de (M.W.); m.millrose@icloud.com (M.M.); 2 Research and Innovation Management (RIM), Biostatistics and Publication of Clinical Trial Studies, Paracelsus Medical University, 5020 Salzburg, Austria; wolfgang.hitzl@pmu.ac.at; 3 Department of Ophthalmology and Optometry, Paracelsus Medical University Salzburg, 5020 Salzburg, Austria; 4 Research Program Experimental Ophthalmology & Glaucoma Research, Paracelsus Medical University, 5020 Salzburg, Austria; 5 Department of Trauma Surgery and Sports Medicine, Garmisch-Partenkirchen Medical Centre, 82467 Garmisch-Partenkirchen, Germany

**Keywords:** osteoarthritis of the knee, therapy of osteoarthritis, intra-articular hyaluronic acid, niacinamide, meniscal damage

## Abstract

**Background:** Osteoarthritis (OA) of the knee is the most common joint disease, characterized by the degeneration of joint cartilage. Intra-articular hyaluronic acid (IAHA) injections are a well-established non-surgical treatment. **Methods:** This retrospective study analyzed knee OA patients receiving IAHA combined with niacinamide injections, assessing pain reduction in relation to patient data, the number of injections, and radiological findings. **Results:** IAHA injections led to significant pain reduction on the numeric rating scale (NRS) (0–10), with a mean decrease of 3.34 ± 1.65. Pain relief was greater with multiple injections. A comparison of subgroups by injection frequency (1, 2, or >2) showed significant pain reduction between 1 and 2 injections (*p* = 0.027) and between 1 and >2 injections (*p* = 0.032). The OA grade measured using the Kellgren–Lawrence (*p* = 0.95) and Vallotton MRI classifications (*p* = 0.50) did not correlate with pain reduction. However, patients with meniscal damage (*p* = 0.02) showed a greater benefit. A strong positive correlation was found between baseline pain intensity and pain reduction (*p* < 0.001; r = 0.61). **Conclusions:** IAHA with niacinamide significantly reduces knee OA pain, with more injections enhancing pain relief. Greater benefits were observed in patients with higher baseline pain and meniscal damage. The favorable safety profile and potential for repeated treatments make IAHA a valuable option in knee OA management.

## 1. Introduction

Osteoarthritis (OA) of the knee is a prevalent and debilitating condition affecting millions of people worldwide, making it the most common joint disease among adults globally [1]. It is a complex, multifactorial disease characterized by the degeneration of joint cartilage and changes in subchondral bone [2]. There are diverse phenotypes, including aetiopathogenic, clinical, and radiographic types, influenced by different pathophysiological mechanisms that drive OA progression [3]. The leading symptoms are pain, reduced mobility, stiffness, and a decreased quality of life. A definitive diagnosis is achieved via radiological imaging [2,4,5].

Epidemiological studies have shown that both exogenous (i.e., macrotrauma, repetitive microtrauma, overweight, joint surgery, and lifestyle) and endogenous (i.e., age, sex, heredity, and post-menopausal changes) risk factors contribute to the development and progression of OA [6]. As Hunter described it, the development of OA is based on excessive mechanical stress in the context of systemic susceptibility [7]. Knee OA is particularly prevalent in individuals who are overweight, with recent studies confirming that excess weight predisposes individuals to OA [8].

There is a strong relationship between meniscal dysfunction and OA, as patients with OA frequently exhibit meniscal abnormalities [9,10]. Dysfunctional menisci contribute to pathological loads on knee cartilage, accelerating its degeneration [11]. Meniscal extrusion (ME), defined as the displacement of the meniscus body beyond the outer margin of the tibial plateau by ≥3 mm, is considered a risk factor for OA [12]. Ghouri et al. demonstrated an association between ME and the structural progression and severity of knee OA, independent of age, sex, and BMI [13]. ME affects knee kinematics and reduces the contact area between the femur and tibia [14,15,16].

Radiography remains the gold standard for diagnosing OA due to its broad accessibility, low cost, and high specificity [17]. Radiographic evaluation of OA primarily focuses on identifying osteophytes, subchondral bone changes, and joint space narrowing, the latter being a key indicator of cartilage loss. Several radiographic classification systems are used to grade OA and monitor disease progression, with the Kellgren–Lawrence (K/L) classification, established in 1957, remaining the most widely accepted standard [18].

Although radiography remains the primary imaging modality, it has limitations, as OA affects the bone while also being a “whole-organ” disorder involving multiple joint tissues, including the synovium, ligaments, subchondral bone, menisci, and surrounding muscles [19,20,21]. Magnetic resonance imaging (MRI) is increasingly important to detect these soft-tissue changes and enhance the visualization of OA features [22]. Synovitis, or inflammation of the synovium, correlates strongly with OA-related pain and is associated with disease progression and severity [23,24]. Bone marrow lesions or bone marrow edema (BME) are also common abnormalities observed in OA [25].

Treatment options for OA are generally divided into three main categories: non-pharmacological (e.g., weight control, exercise, and physiotherapy), pharmacological (e.g., COX-2 inhibitors, non-steroidal anti-inflammatory drugs (NSAIDs), and opioids), and interventional treatments (e.g., intra-articular platelet-rich plasma, corticosteroids, and intra-articular hyaluronic acid (IAHA) injections), as well as surgical options [26,27,28].

Among non-surgical interventions, viscosupplementation with intra-articular hyaluronic acid (IAHA) injections has become a prominent option for reducing pain and improving joint function by restoring the physiological environment of the damaged joint [1]. Hyaluronic acid (HA), a naturally occurring high-molecular-weight glycosaminoglycan in synovial fluid, is critical in maintaining the joint’s viscoelastic properties and lubrication [29]. HA exhibits anti-inflammatory properties by inhibiting inflammation mediators, reducing leukocyte migration, and neutralizing oxygen radicals. It also has analgesic effects by reducing the activity of bradykinin and nociceptive responses [30,31,32]. Exogenous HA can stimulate the synthesis of endogenous HA [33]. Various HA formulations with different molecular weights and structures are available [29]. A relatively new approach involves adding antioxidants, such as vitamin C or vitamin B3, to enhance the therapeutic effects of HA and stabilize the viscosupplement against thermal and oxidative degradation [34]. Niacinamide (vitamin B3) is a component of the enzyme cofactors nicotinamide adenine dinucleotide (NAD+) and nicotinamide adenine dinucleotide phosphate (NADP+), which are important for various physiological processes, including redox reactions. In addition to its anti-inflammatory effects, niacinamide protects HA from hyaluronidase degradation. In an in vitro study, Giardina et al. demonstrated that HA combined with niacinamide exhibited greater resistance to hyaluronidase degradation compared with HA alone [35]. Additionally, Gobbi et al. compared 60 OA patients, with one group receiving IAHA combined with niacinamide (N-HA) and the other receiving IAHA alone. The N-HA group showed longer-lasting effects [36].

There has been a trend toward favorable recommendations for IAHA over the past 20 years, particularly when other treatment options have proven ineffective [5,37,38]. A meta-analysis by Bannuru et al. concluded that the maximal effect of IAHA in the knee joint is achieved at 8 weeks post-injection, with effects detectable for up to 24 weeks [39,40]. Common adverse effects of IAHA include joint swelling and arthralgia, as reported in a 2018 systematic evaluation of patients who received repeated cycles of IAHA over a period of up to 25 months [41].

The primary aim of this observational study was to identify factors influencing pain reduction in knee OA following IAHA with niacinamide. The secondary aim was to assess pain reduction in relation to radiological findings in X-ray and MRI.

## 2. Materials and Methods

### 2.1. Study Population

**Figure 1:**
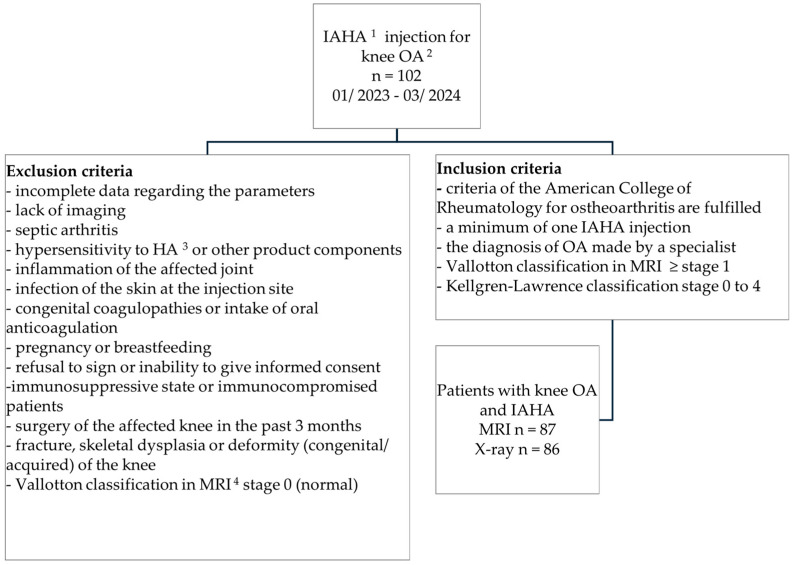
Study flowchart. ^1^ Intra-articular hyaluronic acid; ^2^ osteoarthritis; ^3^ hyaluronic acid; ^4^ magnetic resonance imaging.

This retrospective study analyzed patients with knee OA who received IAHA injections between 1 January 2023 and 31 March 2024 at the outpatient center of a maximum-care hospital. Each patient provided informed consent before enrollment, with the potential benefits and risks of IAHA explained and understood by all participants. In total, 87 of 102 patients with knee OA were included in this study. All underwent MRI, and 86 had knee X-rays. Patients with symptomatic and confirmed knee OA were included if their Vallotton classification was ≥stage 1. All inclusion and exclusion criteria are listed in Figure 1.

### 2.2. Evaluation of Pain and Joint-Specific Variables

The patient-related data collected included age, sex, and body mass index (BMI). Knee pain levels were assessed using the numeric rating scale (NRS) both before the injection and after four weeks. The NRS is a unidimensional, subjective measure of pain intensity ranging from 0 to 10, where 0 indicates no pain, 1–3 mild pain, 4–6 moderate pain, 7–9 severe pain, and 10 the worst possible pain.

The joint-specific variables investigated during the clinical examination included joint stability (categorized as stable or unstable based on the integrity of the anterior cruciate, posterior cruciate, and collateral ligaments) and the range of motion (ROM) of the knee joint. The ROM of this complex hinge joint primarily involves extension and flexion, with a typical range of 0–0–130°. Flexion below 130° was classified as reduced. For the IAHA injections, the number of injections, the intervals between injections, and any side effects observed during follow-up were also recorded.

### 2.3. Radiologic Evaluation of OA

A definitive diagnosis of OA was based on radiological findings. Images were retrospectively evaluated using an approved PACS workstation (Ashvins, Medical Communications, Heidelberg, Germany). X-ray imaging was performed in two planes: anterior–posterior (a.p.) and lateral. The X-ray images were graded according to the Kellgren–Lawrence (K/L) classification, ranging from stage 0 to 4. MRI images were acquired using 1.5 or 3.0 Tesla scanners, with T1-, T2-, and proton-weighted images in the transverse and coronal planes. The MRI grading was performed using the Vallotton classification (Table 1) [42] based on the signal intensity of the cartilage surface, cartilage thickness loss, and bone reaction. Additionally, the images were evaluated for meniscal damage (both medial and lateral lesions) and meniscal extrusion, which was considered significant if the meniscal margin extended ≥3 mm beyond the external aspect of the tibiofemoral compartment. Finally, the number of affected compartments in the joint (medial tibiofemoral, lateral tibiofemoral, and patellofemoral compartments) was recorded.

### 2.4. Hyaluronic Acid

This study used Recosyn^®^ Max forte N HA (Recordati Pharma GmbH, Ulm, Germany). The pre-filled, ready-to-use syringe contained 2 mL of injectable solution, with each dose providing 44 mg of sodium hyaluronate at a high molecular weight of 1.2–2.2 MDa. The HA was linear, specifically indicated for use in larger joints, and approved for single or multiple applications. Additionally, each syringe contained 15 mg/mL of niacinamide. Patients received a single 2 mL injection per appointment, with the option for multiple appointments. Injections were administered under sterile conditions using the anterolateral approach.

### 2.5. Statistical Analysis

The data were checked for consistency and normal distribution using the Kolmogorov–Smirnov test. The Mann–Whitney U and Kruskal–Wallis tests were used for non-parametric variables. Fisher’s exact test was applied for discrete variables in 2 × 2 tables. Cross-tabulation tables larger than 2 × 2 were analyzed using Pearson’s chi-squared test. The Pearson product-moment correlation was used to test associations between two metrically normally distributed variables. Spearman’s rank correlation coefficient was calculated for non-normally distributed variables. Differences in medians were tested using a median test, and the corresponding 95% confidence interval for the difference in medians was calculated. A *p*-value of <0.05 was considered significant. The results are presented as means ± standard deviation and ranges, with the associated minimum and maximum values. All data were obtained and analyzed retrospectively, and statistical analysis was conducted using STATISTICA 13 (Hill, T. and Lewicki, P. Statistics: Methods and Applications. StatSoft, Tulsa, OK, USA [43]) and IBM SPSS Statistics, version 28.0 (IBM, Armonk, NY, USA).

This study was approved by the Institutional Review Committee at Paracelsus Medical University at Nuremberg Hospital (protocol code: IRB-2024-14) per the national legal guidelines.

## 3. Results

### 3.1. Patient-Related Data

The final study population comprised 87 patients. The participants’ baseline characteristics are presented in Table 2. The mean age of the patients was 53.3 ± 14.6 years (range: 16–85), with a mean BMI of 26.2 ± 4.4 kg/m^2^ (range: 17–42). According to the WHO BMI categories, 50.6% (44/87) of participants were classified as overweight (BMI > 24.9). Among the patients, 66.7% (58/87) were male, and 33.3% (29/87) were female. Osteoarthritis (OA) was localized to the right knee in 51.7% of patients, the left knee in 42.5%, and both knees in 5.7%.

Adverse effects were observed in 3.4% (*n* = 3) of patients, all of which were minor: two patients experienced increased pain during movement for more than two days but less than one week, and one patient reported increased pain lasting for three weeks. There was no evidence of infection, and no major complications were detected.

### 3.2. Osteoarthritis Classification

The distribution of patients according to the Kellgren–Lawrence (K/L) and Vallotton classifications showed varying degrees of severity (Table 3). For the K/L classification, among 86 patients, 21.8% were at stage 0, 26.4% at stage 1, 17.2% at stage 2, 27.6% at stage 3, and 5.7% at stage 4. In contrast, the Vallotton classification, assessed for 87 patients, indicated that 1.15% were at stage 1, 20.7% at stage 2, 25.3% at stage 3, and 52.9% at stage 4. Among the 19 patients classified as K/L stage 0, the mean Vallotton stage was 2.79, with only 5.3% (1/19) at Vallotton stage 1. This demonstrates a higher concentration of patients in the most severe stage using the Vallotton classification compared with the K/L classification.

### 3.3. Joint-Specific Variables

The range of motion (ROM) was reduced in 24.1% (21/87) of patients, while knee joint stability was observed in 86.2%. Osteoarthritis (OA) was multi-compartmental in 55.2% of patients. Meniscal damage was present in 86.2% of patients, and meniscal extrusion was observed in 27.6%. Both meniscal lesions and extrusions were more commonly located medially than laterally (Table 4).

### 3.4. Pain Reduction After IAHA Injection

**Figure 2:**
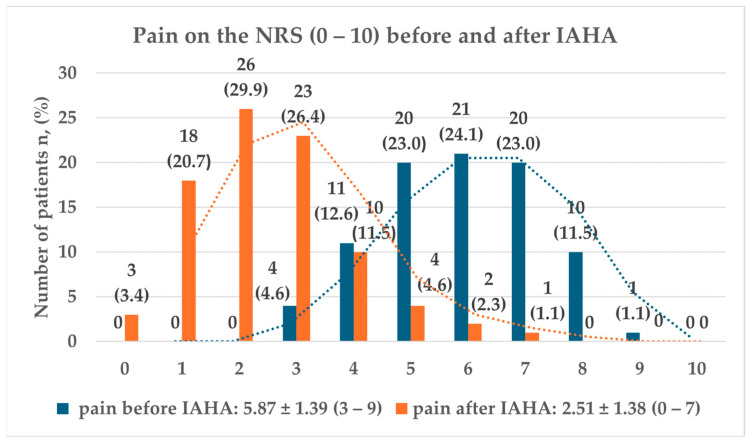
Pain on the numeric rating scale (NRS) before and after intra-articular hyaluronic acid (IAHA) injection.

The average pain score before the injection was 5.87 ± 1.39 on the numeric rating scale (NRS), while at follow-up, the score was 2.51 ± 1.38. The mean reduction in pain on the NRS was 3.34 ± 1.65 (Figure 2).

A correlation analysis of pain reduction following IAHA with niacinamide treatment was conducted in relation to various risk factors (i.e., age and weight), radiological stage, and meniscal involvement. A moderate positive correlation was found between body weight and pain reduction (r = 0.27; *p* = 0.01). However, pain reduction did not reach significance concerning overweight status (*p* = 0.09). Age also showed a moderate correlation with pain reduction (r = 0.21; *p* = 0.05). A moderate-to-strong positive correlation was identified between the pain intensity before injection and the subsequent pain reduction (r = 0.61; *p* < 0.001). No significant correlation was observed between pain reduction and the osteoarthritis stage, as classified by either the Kellgren–Lawrence (KL) (*p* = 0.95) or Vallotton (*p* = 0.50) classifications. Additionally, pain reduction was not significantly correlated with the affected knee joint compartment (medial/lateral/patellofemoral) (*p* = 0.93) or whether the osteoarthritis was uni- or multi-compartmental (*p* = 0.48). Among patients without meniscal damage, the median pain reduction was 2, while for those with meniscal damage, the median reduction was 3, resulting in a median difference of 1 unit (95% CI: 0–2; *p* = 0.019). The median pain reduction was 0.8 points higher on the NRS for patients with medial meniscal lesions compared with those with lateral lesions (*p* < 0.05). There was no correlation between pain reduction and the presence of meniscal extrusion (*p* = 0.82).

### 3.5. Number of Injections

**Figure 3:**
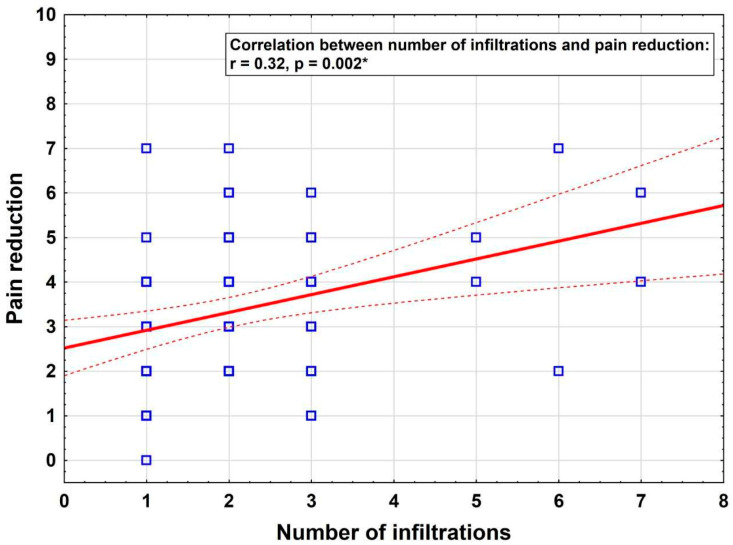
Correlation between number of infiltrations and pain reduction. The asterisk (*) represents the *p*-value of the statistical test. One asterisk = *p* < 0.05.

The number of injections ranged from 1 (40.2%) to 7 (2.3%), with a mean of 2.06 (Table 5). In total, this study included 179 infiltrations. Thirty-five patients received one injection, thirty patients received two injections, and twenty-two patients received more than two injections. The mean interval until the next treatment was 2.25 ± 2.17 months (range: 1–12). A low-to-moderate positive correlation was found between the number of infiltrations and pain reduction (r = 0.32; *p* = 0.002) (Figure 3). Each additional injection was associated with a reduction of 0.4 points on the NRS.

### 3.6. Pain Reduction Varies with Number of Infiltrations

**Figure 4:**
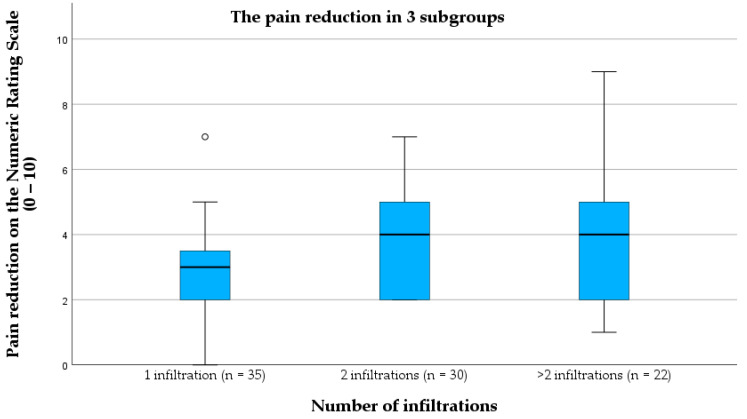
The pain reduction after 1, 2, or more than 2 infiltrations on the numeric rating scale.

The pain reduction was compared across three subgroups based on the number of infiltrations: one infiltration (*n* = 35), two infiltrations (*n* = 30), and more than two infiltrations (*n* = 22). The pain reduction differed significantly between the three groups (*p* = 0.009). A single infiltration reduced pain by a median of 3 points on the NRS. The median pain reduction was four for both two and more than two infiltrations, with a wider interquartile range than that seen with only one infiltration. The 1-infiltration group showed a lower range of pain reduction, while the >2-infiltration group exhibited the widest range, with some patients achieving substantial pain relief (up to 9 points on the NRS) (Figure 4).

Pairwise comparisons revealed significant differences between the groups: one vs. two infiltrations (*p* = 0.027) and one vs. more than two infiltrations (*p* = 0.032), while the difference between two and more than two infiltrations was not significant (*p* > 0.05).

## 4. Discussion

This study analyzed a database of patients with knee OA who received IAHA injections containing niacinamide, examining patient-related factors, joint-specific variables, and radiologic measurements. The findings provide insights into the efficacy of IAHA injections and highlight factors that may influence treatment outcomes. The main conclusions of this study are that IAHA with niacinamide provided significant pain reduction, especially with more than one injection; the OA grade in radiological imaging did not correlate with pain reduction; and patients with higher baseline pain levels and meniscal damage experienced greater benefits from the injection.

The primary outcome of the study was pain reduction, measured using the numeric rating scale (NRS). The mean pain reduction on the NRS (0–10) was significant at 3.34 ± 1.65 (range: 0–9). This finding aligns with previous research indicating that IAHA injections can provide modest but meaningful pain relief for patients with knee OA. The Cochrane review Viscosupplementation for the Treatment of Osteoarthritis of the Knee by Bellamy et al. [44] of 76 randomized controlled trials showed a modest statistically significant effect on pain reduction compared with the placebo, with a standardized mean difference (SMD) of 0.37, indicating that pain reduction increased from a baseline of 28% to 54%. Similarly, Ye, Ko et al. analyzed the visual analog scale (VAS) in patients with K/L grades 2 to 3 OA one week after IAHA injections and showed a significant decrease from 75.2 ± 14.7 to 44.2 ± 17.2 (*p* < 0.05) [45], comparable to the reduction of over 3 points on the NRS observed in this study. This reduction in pain enabled patients to decrease their use of pharmacological therapies, thereby reducing the potential for adverse effects. IAHA administration, especially for older patients or those with a higher risk profile, offers a safe alternative to long-term NSAID therapy.

A critical finding in this study was the relationship between the number of IAHA injections and pain reduction. Pain relief was greater among patients receiving multiple injections. A pairwise comparison of the subgroups divided by the number of infiltrations (1, 2, or >2) showed significant pain reduction between 1 and 2 injections (*p* = 0.027) as well as between 1 and more than 2 injections (*p* = 0.032). Specifically, patients who received one injection reported a median pain reduction of 3 points on the NRS, while those who received two or more injections experienced a median pain reduction of 4 points. This finding aligns with the systematic review and meta-analysis by Concoff et al., which demonstrated the largest effect size for pain relief after 2–4 IAHA injections compared with intra-articular saline, while a single injection showed no significant pain relief compared with saline [46]. Although the difference in pain reduction between the 2-injection and >2-injection groups was not statistically significant (*p* > 0.05), the trend suggests that there may be diminishing returns with each additional injection. The moderate positive correlation between the number of injections and pain reduction (r = 0.32; *p* = 0.002) indicates that, on average, additional injections provide further relief, though the benefits might plateau after a certain point.

The risk factors for OA, the radiological grade, and meniscal pathologies were analyzed to understand their impacts on pain reduction. Pain reduction did not correlate with the stage of osteoarthritis measured using the Kellgren–Lawrence classification (*p* = 0.95) or the Vallotton classification (*p* = 0.50). This suggests that the radiological severity of OA may not predict the degree of pain relief from IAHA injections and that IAHA may also be effective in higher-grade OA. A moderate positive correlation (r = 0.27; *p* = 0.01) was found between body weight and pain reduction, suggesting that heavier patients experienced greater pain relief. However, the overweight status itself did not show a significant correlation (*p* = 0.09). This could indicate that the absolute weight status, rather than the relative weight status (overweight vs. not overweight), plays a role in the response to IAHA injections. The presence of meniscal lesions, particularly medial lesions, was associated with greater pain reduction, with a median difference of 1 point on the numeric rating scale (NRS) compared with patients without meniscal damage (*p* = 0.019). This finding suggests that meniscal involvement may exacerbate pain and potentially influence the efficacy of IAHA injections. However, no significant correlation was found between pain reduction and the presence of meniscal extrusion (*p* = 0.82), indicating that extrusion might not significantly affect the outcome of IAHA treatment.

There was a strong positive correlation between initial pain intensity and pain reduction (r = 0.61; *p* < 0.001). Patients with higher baseline pain levels experienced greater absolute reductions in pain, suggesting that those suffering from more intense pain initially may derive more noticeable benefits from IAHA injections. However, the risk of response bias due to higher expectations among patients with higher pain levels should be considered. Despite this, Pelletier et al. also identified high levels of knee pain as a predictive determinant of IAHA treatment benefit [47], supporting the idea that patients with more severe pain may experience more significant improvements with IAHA therapy.

The injected HA in this study contained niacinamide, which has previously been shown to have longer-lasting effects (up to six months) compared with HA without niacinamide [36]. The follow-up for pain reduction evaluation in this study was conducted 4 weeks post-injection. Future research should extend the follow-up period to 3 to 6 months post-injection to better assess the duration of IAHA’s efficacy when combined with niacinamide. Additionally, the increased efficiency of HA due to the positive characteristics of niacinamide should be further explored in studies that include comparison groups to better understand its potential benefits and confirm its role in enhancing the effects of HA injections.

Additionally, the effects of niacinamide should be further investigated alongside other antioxidants that could be considered as potential therapeutic options for OA. Roman-Blas et al. demonstrated that N-acetylcysteine (NAC) reduced the synthesis of catabolic mediators, such as matrix metalloproteinases, nitric oxide, and prostaglandin E2, in osteoarthritic synoviocytes. Consequently, NAC has shown potent antioxidant and anti-catabolic effects in patients with OA [48].

Following the algorithm recommended by the European Society for Clinical and Economic Aspects of Osteoporosis and Osteoarthritis (ESCEO), IAHA is recommended for patients who remain symptomatic after advanced pharmacological management, including intermittent or continuous cycles of oral NSAIDs [49]. The European League of Rheumatism (EULAR) recommends IAHA therapy with a level of evidence of 1a or 1b [50]. IAHA demonstrates moderate benefits when comparing these guidelines with the observed efficacy of IAHA injections in this and previous studies. While it may not be the ultimate solution for osteoarthritis, it can provide clinically relevant relief for certain patients. In contrast, the Osteoarthritis Research Society International (OARSI) guidelines do not recommend the use of IAHA [51]. An important criticism of current guidelines is the potential for adverse effects associated with intra-articular injections. However, in this study, the safety profile of IAHA injections was favorable, with only 3.4% of patients experiencing minor complications, such as transient pain. No major complications or infections were reported, suggesting that IAHA is a safe and viable treatment option for managing knee OA in patients who do not respond adequately to other therapies.

In this study, a discrepancy in the severity of OA was observed between the two classifications. According to the Kellgren–Lawrence (K/L) classification, 21.8% of the 86 patients were at stage 0, indicating no abnormality, while only 27.6% were at stage 3 and 5.7% at stage 4. This suggests that only one-third of the patients had moderate-to-severe OA. In contrast, the Vallotton classification indicated that 25.3% of patients were at stage 3 and 52.9% at stage 4, demonstrating a higher concentration of patients at more severe stages in the Vallotton classification compared with the K/L classification.

The 19 patients classified as K/L stage 0 had a mean Vallotton score of 2.79, suggesting that the Vallotton classification may be more sensitive to certain aspects of joint pathology, such as cartilage integrity and subchondral bone changes. This discrepancy highlights a potential limitation of the K/L classification in the early stages of the disease, where radiographic imaging may not adequately reflect the extent of cartilage damage. As a result, patients in the earlier stages of OA may not receive optimal therapeutic options based on X-ray findings alone. This emphasizes the importance of using more sensitive imaging techniques, such as MRI, for a comprehensive evaluation of joint health, especially in the early stages of OA.

### Limitations

This study had several limitations that must be considered when interpreting the results. Due to its retrospective design, potential sources of bias, such as selection bias, recall bias, and the lack of randomization, could have affected the findings. The absence of a randomized control group further limits our ability to draw causal conclusions about the effectiveness of IAHA injections. Additionally, the follow-up duration was relatively short, and long-term outcomes were not assessed, which limits the understanding of the sustained effects of IAHA treatment over time.

The use of the numeric rating scale (NRS) to assess pain reduction, while widely used, is a subjective measure and may be influenced by reporting biases or external factors. Standardized questionnaires, such as the Oxford Knee Score, could provide a more comprehensive and comparable assessment of knee function and pain relief. Moreover, this study did not collect information on the duration of pain relief or any functional improvements following treatment, which would have been valuable for understanding the long-term benefits of IAHA.

Despite these limitations, this study has notable strengths. The primary focus on pain reduction provides valuable insights into the immediate benefits of IAHA, particularly in relation to meniscal pathologies, which have not been extensively explored in previous research. Additionally, the analysis of MRI and X-ray images strengthens this study, as it provides a detailed view of the joint health and pathology in the cohort, helping to correlate imaging findings with clinical outcomes. Overall, despite its limitations, this study contributes valuable information on the potential efficacy of IAHA in knee OA, particularly in patients with meniscal damage.

## 5. Conclusions

In conclusion, IAHA injections with niacinamide appear to be an effective treatment for pain relief in knee osteoarthritis (OA), with the number of injections playing a significant role in the degree of pain reduction. While a single injection can provide moderate pain relief, multiple injections, especially those exceeding two, lead to more substantial symptom improvement, although with a broader range of responses. The benefits were more pronounced in patients with higher baseline pain levels and those with meniscal damage, suggesting that these factors influence the treatment’s effectiveness. Furthermore, patients with higher OA grades, assessed via radiological imaging, and advanced joint degeneration also experienced notable improvements.

Given these promising results, further research is recommended to investigate the long-term effects and potential advantages of combining hyaluronic acid with niacinamide. This study contributes to the growing body of literature supporting the efficacy and safety of IAHA injections for knee OA, emphasizing the importance of individualized treatment approaches in managing this chronic and debilitating condition. The findings suggest that IAHA, particularly when combined with niacinamide, may provide an effective option for pain relief and functional improvement in knee OA patients, helping to enhance their quality of life while minimizing the need for more invasive treatments.

## Figures and Tables

**Table 1:** The Kellgren–Lawrence classification for staging OA in radiography [18] versus the Vallotton classification for grading articular lesions in MRI [42].

Stage	Kellgren–Lawrence Classification	Vallotton Classification
**0**	Normal joint with no detectable osteophytes, joint space narrowing, sclerosis, or deformity	Normal joint with intact cartilage, no bone marrow lesions, no synovitis, and normal menisci
**1**	Incipient osteoarthritis, no osteophytes, no definite joint space narrowing, and mild subchondral sclerosis	Cartilage surface intact and hypo- or hyper-signal
**2**	Beginning osteophytes, moderate joint space narrowing, and moderate subchondral sclerosis	Mild surface irregularity and/or focal loss < 50% thickness of cartilage, small osteophytes, and mild synovitis
**3**	>50% joint space narrowing, rounded femoral condyle, extensive subchondral sclerosis, and extensive osteophyte formation	Severe surface irregularity, focal loss > 50% thickness, no subchondral edema, bone intact, moderate osteophytes and synovitis, and meniscal damage or extrusion
**4**	Joint destruction, obliterated joint space, and subchondral cysts in the tibial head and femoral condyle	Subchondral edema, bone reaction, and meniscal damage or extrusion

**Table 2:** Patient-related data.

Age, y (Mean ± SD, Range)	53.3 ± 14.6 (16–85)
Gender, female (*n*, %)	29 (33.3%)
Gender, male (*n*, %)	58 (67.7%)
BMI (mean ± SD, range)	26.2 ± 4.4 (17–42)
Adverse effects (*n*, %)	3 (3.4%)

**Table 3:** Kellgren–Lawrence (K/L) and Vallotton classifications.

Stage	Kellgren–Lawrence (*n* = 86)	Vallotton (*n* = 87)
0	19 (21.8%)	
1	23 (26.4%)	1 (1.15%)
2	15 (17.2%)	18 (20.7%)
3	24 (27.6%)	22 (25.3%)
4	5 (5.7%)	46 (52.9%)

**Table 4:** Number of affected joint compartments (medial tibiofemoral, lateral tibiofemoral, patellofemoral) and meniscal involvement.

Pathology	Yes	No
Multi-compartmental	55.2% (48/87)	44.8% (39/87)
Meniscal damage	86.2% (75/87)	13.8% (12/87)
Lesion of the medial meniscus	56.3% (49/87)	
Lesion of the lateral meniscus	16.1% (14/87)	
Lesion of the medial and lateral menisci	13.8% (12/87)	
Meniscal extrusion	27.6% (24/87)	72.4% (63/87)
Medial meniscal extrusion	19.5% (17/87)	
Lateral meniscal extrusion	5.7% (5/87)	
Medial and lateral extrusion	2.3% (2/87)	

**Table 5:** The number of intra-articular hyaluronic acid (IAHA) injections, with a mean of 2.06.

Number of Injections	Patients (*n* = 87)	
1	35	40.2%
2	30	34.5%
3	16	18.3%
4	0	0%
5	2	2.3%
6	2	2.3%
7	2	2.3%

## Data Availability

Data are contained within the article.
